# Inhibition of unfolded protein response prevents post‐anesthesia neuronal hyperactivity and synapse loss in aged mice

**DOI:** 10.1111/acel.13592

**Published:** 2022-03-17

**Authors:** Kai Chen, Qiuping Hu, Riya Gupta, Jessie Stephens, Zhongcong Xie, Guang Yang

**Affiliations:** ^1^ Department of Anesthesiology Columbia University Irving Medical Center New York New York USA; ^2^ Barnard College of Columbia University New York New York USA; ^3^ New York Medical College Valhalla New York USA; ^4^ Geriatric Anesthesia Research Unit, Department of Anesthesia, Critical Care and Pain Medicine Massachusetts General Hospital and Harvard Medical School Charlestown Massachusetts USA

**Keywords:** delirium, dendritic spine, neuronal activity, sevoflurane, synapse, unfolded protein response

## Abstract

Delirium is the most common postoperative complication in older patients after prolonged anesthesia and surgery and is associated with accelerated cognitive decline and dementia. The neuronal pathogenesis of postoperative delirium is largely unknown. The unfolded protein response (UPR) is an adaptive reaction of cells to perturbations in endoplasmic reticulum function. Dysregulation of UPR has been implicated in a variety of diseases including Alzheimer's disease and related dementias. However, whether UPR plays a role in anesthesia‐induced cognitive impairment remains unexplored. By performing in vivo calcium imaging in the mouse frontal cortex, we showed that exposure of aged mice to the inhalational anesthetic sevoflurane for 2 hours resulted in a marked elevation of neuronal activity during recovery, which lasted for at least 24 hours after the end of exposure. Concomitantly, sevoflurane anesthesia caused a prolonged increase in phosphorylation of PERK and eIF2α, the markers of UPR activation. Genetic deletion or pharmacological inhibition of PERK prevented neuronal hyperactivity and memory impairment induced by sevoflurane. Moreover, we showed that PERK suppression also reversed various molecular and synaptic changes induced by sevoflurane anesthesia, including alterations of synaptic NMDA receptors, tau protein phosphorylation, and dendritic spine loss. Together, these findings suggest that sevoflurane anesthesia causes abnormal UPR in the aged brain, which contributes to neuronal hyperactivity, synapse loss and cognitive decline in aged mice.

## Graphical abstract

Older individuals are particularly susceptible to postoperative brain dysfunction, but the mechanism underlying anesthesia‐induced cognitive decline is not yet clear. This study shows that exposure of aged mice to anesthesia causes an abnormal increase in neuronal activity in the frontal cortex where dysregulation of unfolded protein response (UPR) is also observed. Inhibition of PERK, a key component of UPR, prevents anesthesia‐induced neuronal hyperactivity, synapse loss, and memory impairment.

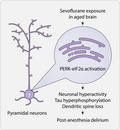

**Abbreviations**
AMPA: α‐amino‐3‐hydroxy‐5‐methyl‐4‐isoxazolepropionic acid
EIF2α: eukaryotic translation initiation factor 2α
ER: endoplasmic reticulum
NMDA: N‐methyl‐D‐aspartate
PERK: protein kinase RNA‐like endoplasmic reticulum kinase
Sevo: sevoflurane
Tau: microtubule‐associated protein
UPR: unfolded protein response

## 1 INTRODUCTION

Delirium is a neuropsychiatric syndrome characterized by a sudden onset of mental confusion and emotional disruption (Inouye et al., 2014; Lipowski, 1989; Morandi et al., 2014; Wilson et al., 2020). Postoperative delirium is a form of delirium that manifests in patients who have experienced anesthesia during surgical procedures. It usually occurs within days after anesthesia and surgery and is associated with accelerated cognitive decline, development of Alzheimer's disease and related dementia (ADRD), and high rates of morbidity and mortality (Davis et al., 2017; Fong et al., 2009; McCusker et al., 2002; Rudolph & Marcantonio, 2011; Sieber, 2009; Sprung et al., 2017). Although the elderly are much more vulnerable in developing postoperative delirium compared with younger populations, with rates as high as 60% in the hospital (Cole et al., 2003; Kotekar et al., 2014), the neuropathogenesis of postoperative delirium remains mostly unknown.

An increasing number of preclinical studies suggest that prolonged exposure to general anesthesia can induce delirium‐like behavior, including cognitive impairment, as well as molecular and cellular changes characteristic of neurodegeneration such as endoplasmic reticulum (ER) stress, tau hyperphosphorylation, neuroinflammation, and cell death (Vutskits & Xie, 2016). For example, young mice repeatedly exposed to sevoflurane, a widely used inhalational anesthetic, exhibit abnormal social and impulsive behavior, deficits in motor learning, and impairments in fear and spatial memory (Le Freche et al., 2012; Satomoto et al., 2009; Xie et al., 2020; Yu et al., 2020). In neonatal rodents, exposure to sevoflurane causes a transient increase in neuronal activity, rapid changes of dendritic spine density, and elevation of tau protein phosphorylation and interleukin‐6 expression in the brain (Dong et al., 2021; Lu et al., 2017; Tao et al., 2014; Yang et al., 2020; Yu et al., 2020; Zhang et al., 2020). Compared with younger adults, anesthesia induces greater delirium‐like behavior in older animals (Kilicaslan et al., 2013; Liufu et al., 2020; Wiklund et al., 2009). However, little is known about the effects of sevoflurane treatment on neuronal function and synaptic plasticity in the aged brain.

Aging is associated with changes in the unfolded protein response (UPR), a signaling reaction triggered by the ER stress (Hetz & Saxena, 2017). The UPR is initiated via the activation of three sensor proteins in the ER membrane: inositol‐requiring enzyme 1, activating transcription factor 6, and the PKR‐like ER kinase (PERK). As a key component of the UPR, PERK activation induces the phosphorylation of eukaryotic initiation factor 2α (eIF2α), a translational factor that controls the initiation step of protein synthesis. Phosphorylation of eIF2α inhibits global protein synthesis, thereby protecting cells from an overload of proteins in the ER lumen. However, under conditions where ER stress is sustained, prolonged activation of UPR results in impaired synaptic plasticity and cell death (Freeman & Mallucci, 2016; Kim et al., 2006). Abnormally hyperphosphorylated PERK and eIF2α have been observed in ADRD and other neurodegenerative diseases (Hughes & Mallucci, 2019), but whether PERK/eIF2a signaling contributes to neurocognitive dysfunction induced by anesthesia remains unknown.

In this study, we examined the cortical effects of sevoflurane anesthesia in young adult (4 months) and aged (>18 months) mice. Sevoflurane is one of the most used anesthetics in clinical practice and has been associated with postoperative cognitive impairment in patients (Geng et al., 2017). Using in vivo Ca^2+^ imaging, we found that sevoflurane exposure caused a marked increase in neuronal activity during recovery in the frontal association cortex of aged but not young mice. This elevation of neuronal activity lasted for at least 24 h after the end of exposure and was paralleled by the increased phosphorylation of PERK and eIF2α in frontal cortical neurons. Genetic deletion or pharmacological inhibition of PERK reduced sevoflurane‐induced frontal hyperexcitability and cognitive impairment. In addition, PERK suppression restored synaptic protein expression and tau phosphorylation, and reversed anesthesia‐induced synapse loss and cell death in the aged cortex. These results underscore the importance of UPR dysfunction in anesthesia‐induced neurocognitive dysfunction in aged mice.

## 2 RESULTS

### 2.1 Sevoflurane induces post‐anesthesia neuronal hyperactivity in the aged brain

**FIGURE 1:**
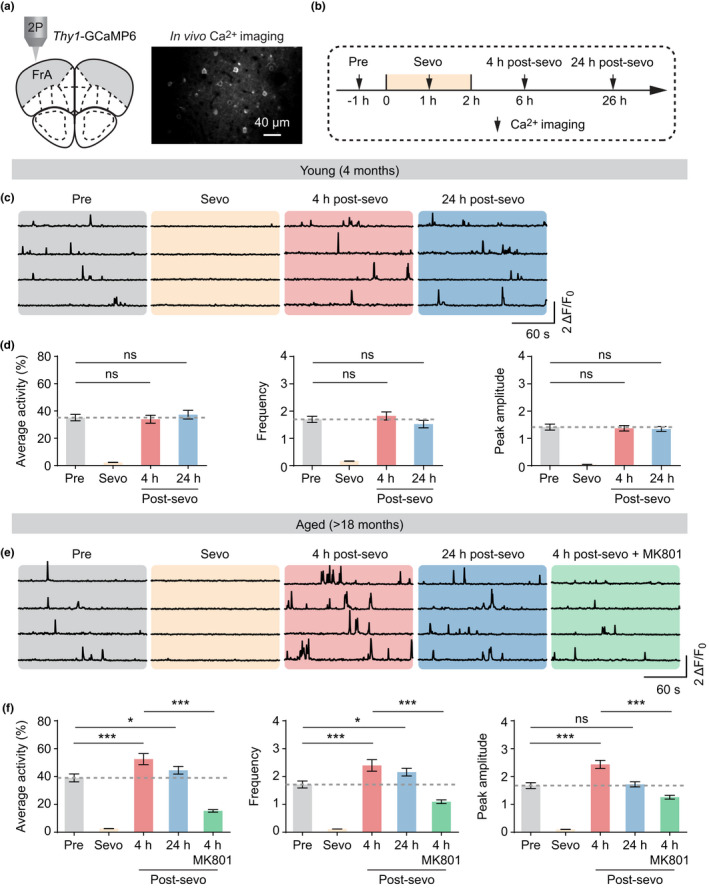
Neuronal hyperactivity in the frontal cortex of aged mice after sevoflurane anesthesia. (a) Left, schematic showing in vivo two‐photon (2P) Ca^2+^ imaging in the frontal association cortex (FrA) of *Thy1*‐GCaMP6s mice. Right, representative image of L5 pyramidal neurons expressing GCaMP6s. (b) Experimental timeline. (c) Representative Ca^2+^ traces in young mice before, during, and 4 and 24 h after sevoflurane anesthesia. (d) Average integrated activity, frequency, and peak amplitude of Ca^2+^ transients in young mice before, during, and after sevoflurane exposure (*n* = 4 mice; average activity, *F* (3, 733) = 67.20, *p* < 0.0001, frequency, *F* (4, 829) = 48.84, *p* < 0.0001; amplitude, *F* (4, 829) = 64.53, *p* < 0.0001). (e) Representative Ca^2+^ traces in aged mice before, during, and 4 and 24‐h post‐anesthesia. MK801 was locally applied to the FrA at 4 h post‐anesthesia. (f) Average integrated activity, frequency, and peak amplitude of Ca^2+^ transients in aged mice before, during, and 4 and 24‐h post‐anesthesia (*n* = 4 mice; average activity, *F* (4, 959) = 73.19, *p* < 0.0001; frequency, *F* (4, 961) = 43.75, *p* < 0.0001; amplitude, *F* (4, 958) = 91.99, *p* < 0.0001). Summary data are presented as mean ± *SEM*. ns, not significant; *p < 0.05, **p < 0.01, ***p < 0.001 by one‐way ANOVA followed by Bonferroni's test

To determine the cortical effects of sevoflurane anesthesia, we performed in vivo two‐photon Ca^2+^ imaging in the frontal association cortex of young adult and aged mice (Figure 1a). Transgenic mice expressing the genetically encoded calcium indicator GCaMP6s in layer 5 (L5) pyramidal neurons were used to measure neuronal activity before, during and after 2‐h sevoflurane anesthesia (Figure 1b) (Cichon et al., 2020). As expected, the level of somatic Ca^2+^ in frontal pyramidal neurons was greatly reduced following anesthesia induction in both young and aged mice as compared to the pre‐anesthesia baseline (i.e., resting awake) (young, Δ*F*_sevo_/Δ*F*_bl_: 0.06; aged, Δ*F*_sevo_/Δ*F*_bl_: 0.07) (Figure 1c,d,e,f). This anesthesia‐induced suppression of neuronal activity was quickly restored during anesthesia recovery. At 4‐h post‐anesthesia, neuronal Ca^2+^ activity in young mice was similar to the basal activity before anesthesia (Figure 1c,d). To our surprise, we found the level of somatic Ca^2+^ in the frontal cortex of aged mice was significantly increased during anesthesia recovery, exceeding the basal activity before sevoflurane exposure (Figure 1e,f). At 4‐h post‐anesthesia, the average integrated activity (52.51 ± 3.99 vs. 39.0 ± 2.82, *p* < 0.0001), frequency (2.34 ± 0.21 vs. 1.71 ± 0.12, *p* < 0.0001) and peak amplitude (2.44 ± 0.14 vs. 1.67 ± 0.11, *p* < 0.0001) of Ca^2+^ transients in aged mice were approximately 1.4‐fold of the pre‐anesthesia baseline. This increase in frontal Ca^2+^ lasted for at least 24 h in aged mice (Figure 1e,f). Local application of MK801 (100 µM), an NMDA receptor antagonist, at 4‐h post‐anesthesia significantly reduced the level of somatic Ca^2+^ (Figure 1e,f), suggesting that the increase in Ca^2+^ transients during recovery is mediated by NMDA receptors. Together, these findings indicate that sevoflurane causes neuronal hyperactivity in the frontal cortex of aged mice, but not young mice.

### 2.2 Sevoflurane activates PERK‐eIF2α signaling in the aged brain

**FIGURE 2:**
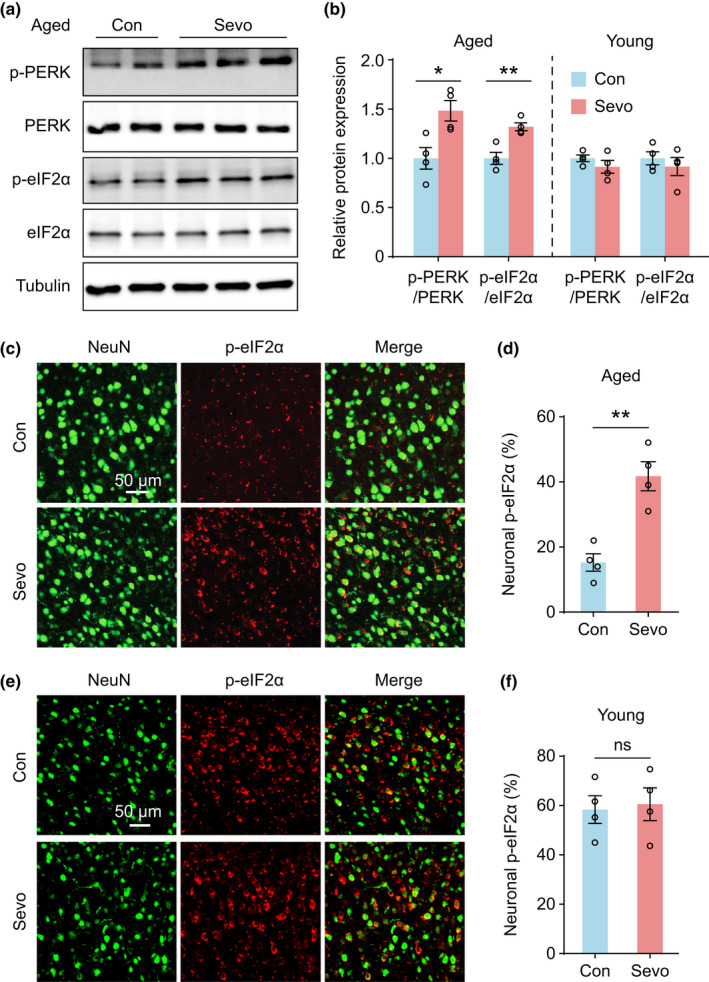
Sevoflurane anesthesia activates PERK/eIF2α in the frontal cortex of aged mice. (a) Representative image of Western blotting in the frontal cortex of aged mice. (b) Quantification of p‐PERK and p‐eIF2α in young and aged mice 4 h after sevoflurane anesthesia (*n* = 4 mice per group; aged, p‐PERK/PERK, *t*_6_ = 3.207, *p* = 0.018; p‐eIF2α/eIF2α, *t*_6_ = 4.401, *p* = 0.005; young, p‐PERK/PERK, *t*_6_ = 1.171, *p* = 0.286; p‐eIF2α/eIF2α, *t*_6_ = 0.742, *p* = 0.486). (c) Representative coronal sections of the aged frontal cortex stained with a neuronal marker NeuN and a phosphorylated eIF2α antibody 24 h after sevoflurane exposure. Scale bar, 50 µm. (d) Quantification of p‐eIF2α expression in the frontal neurons of aged mice (*n* = 4 mice per group, *t*_6_ = 5.088, *p* = 0.002). (e) Immunostaining of NeuN and p‐eIF2α in the frontal cortex of young mice 24 h after sevoflurane exposure. Scale bar, 50 µm. (f) Quantification of p‐eIF2α expression in the frontal neurons of young mice (*n* = 4 mice each group, *t*_6_ = 0.248, *p* = 0.812). Summary data are presented as mean ± *SEM*. ns, not significant; *p < 0.05, **p < 0.01 by unpaired, two‐tailed student's *t* test

Previous studies have shown that exposure to sevoflurane could cause ER stress in the developing brain (Liu et al., 2017; Shen et al., 2018). Given our findings that sevoflurane‐induced age‐dependent changes in frontal neuronal activity, we subsequently asked whether sevoflurane could cause age‐dependent changes in the ER stress response. To this end, we examined the phosphorylation of PERK and eIF2α in the frontal cortex of young and aged mice after 2‐h sevoflurane exposure. Western blot analysis showed that the protein levels of phospho‐PERK and phospho‐eIF2α (Ser51) were significantly increased in the brain of aged but not young animals 4 h after sevoflurane anesthesia (Figure 2a,b), suggesting age‐related differences in the activation of PERK‐eIF2α pathway. At 24‐h post‐anesthesia, the phosphorylation of eIF2α in frontal cortical neurons remained elevated, as evidenced by the immunofluorescence staining of phospho‐eIF2α (Figure 2c,d). By contrast, there were no significant changes in the amount of phospho‐eIF2α in the frontal cortex of young mice before and 24 h after sevoflurane anesthesia (Figure 2e,f). These results indicate that sevoflurane anesthesia causes protracted activation of PERK‐eIF2α signaling in the frontal cortex of aged, but not young mice.

### 2.3 Genetic deletion and pharmacological inhibition of PERK prevents sevoflurane‐induced neuronal hyperactivity

**FIGURE 3:**
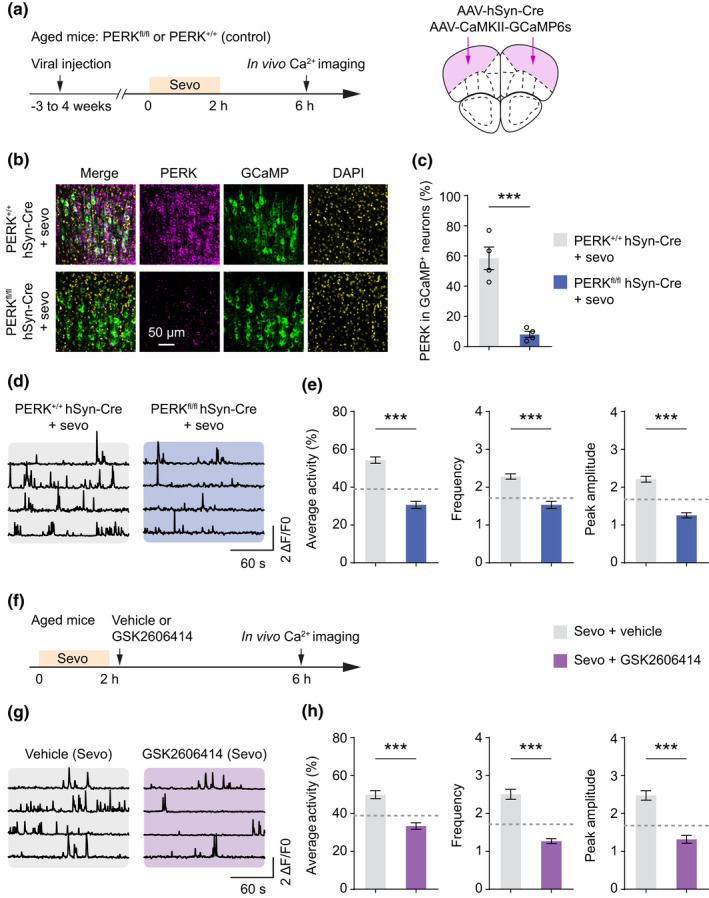
Genetic or pharmacological inhibition of PERK attenuates sevoflurane‐induced neuronal hyperactivity in aged mice. (a) Experimental timeline for genetic inhibition of PERK, sevoflurane treatment, and in vivo Ca^2+^ imaging in aged mice. AAV‐hSyn‐Cre and AAV‐CaMKII‐GCaMP6s were bilaterally injected into the frontal cortex of PERK^fl/fl^ mice to conditionally knockout PERK and express GCaMP6s. (b) Immunostaining of PERK in the frontal cortex 3–4 weeks after viral infection. Scale bar, 50 µm. (c) Quantification of PERK expression in GCaMP‐expressing pyramidal neurons (*n* = 4 mice per group, *t*_6_ = 6.485, *p* = 0.0006). (d) Representative Ca^2+^ traces from PERK^+/+^ hSyn‐Cre and PERK^fl/fl^ hSyn‐Cre mice 4 h after sevoflurane anesthesia. (e) Quantification of average integrated activity, frequency, and peak amplitude of Ca^2+^ transients in PERK^+/+^ hSyn‐Cre and PERK^fl/fl^ hSyn‐Cre mice 4‐h post‐anesthesia (*n* = 4 mice per group; average activity, *t*_433_ = 9.184, *p* < 0.0001; frequency, *t*_433_ = 6.489, *p* < 0.0001; amplitude, *t*_433_ = 8.538, *p* < 0.0001). Dashed line indicates pre‐anesthesia baseline. (f) Experimental timeline for pharmacological inhibition of PERK. Aged mice were orally administrated GSK2606414 (50 mg/kg), a PERK inhibitor, after sevoflurane anesthesia. (g) Representative Ca^2+^ traces at 4‐h post‐anesthesia in aged mice treated with vehicle or GSK2606414. (h) Quantification of average integrated activity, frequency, and peak amplitude of Ca^2+^ transients in aged mice treated with vehicle or GSK2606414 after sevoflurane anesthesia (*n* = 4 mice each group; average activity, *t*_434_ = 5.546, *p* < 0.0001; frequency, *t*_432_ = 8.602, *p* < 0.0001; amplitude, *t*_420_ = 6.645, *p* < 0.0001). Dashed line indicates pre‐anesthesia baseline. Summary data are presented as mean ± *SEM*. ***p < 0.001 by unpaired, two‐tailed student's *t* test

To determine the relationship between sevoflurane‐induced neuronal hyperactivity and PERK/eIF2α hyperphosphorylation in aged mice, we used Cre‐Lox recombination technology to genetically delete PERK in the frontal cortex. To achieve selective deletion of PERK from frontal cortical neurons, we bilaterally injected AAV‐hSyn‐Cre into the frontal cortex of aged PERK^fl/fl^ mice or PERK^+/+^ mice (control) (Figure 3a). AAV‐CaMKII‐GCaMP6s was injected simultaneously to enable the expression of GCaMP6s in pyramidal cells. Three to four weeks after viral injection, we verified the efficacy of PERK deletion by immunofluorescence staining (Figure 3b). We observed PERK immunofluorescence in ~8% pyramidal neurons of PERK^fl/fl^ hSyn‐Cre AAV mice (Figure 3c), and no difference was detected in the total number of NeuN^+^ cells between groups (Figure S1a,b). We proceeded to perform in vivo Ca^2+^ imaging in the frontal cortex of PERK^fl/fl^ hSyn‐Cre AAV mice and PERK^+/+^ hSyn‐Cre AAV mice before and after sevoflurane anesthesia (Figure 3a). We found that selective PERK deletion in frontal neurons had no effects on the basal activity of pyramidal neurons before sevoflurane exposure (Figure S1c,d) but attenuated post‐anesthesia neuronal hyperactivity (Figure 3d,e, Figure S2a). Four hours after sevoflurane anesthesia, the average integrated activity, frequency, and peak amplitude of Ca^2+^ transients were significantly decreased in PERK^fl/fl^ hSyn‐Cre AAV mice as compared to PERK^+/+^ hSyn‐Cre AAV control mice. These results indicate that genetic deletion of PERK attenuates sevoflurane‐induced frontal hyperexcitability in aged mice.

To confirm our findings that PERK‐eIF2α signaling disruption in the frontal cortex reverses post‐anesthesia neuronal hyperactivity, we administered aged mice GSK2606414 (50 mg/kg, oral gavage), a highly selective PERK inhibitor (Moreno et al., 2013), following sevoflurane anesthesia (Figure 3f, Figure S3a,b). Consistent with genetic deletion, pharmacological inhibition of PERK potently reduced the frontal neuronal hyperactivity at 4 h post‐anesthesia (Figure 3g,h, Figure S2b). Combined, these results indicate that suppression of PERK activity in aged mice prevents sevoflurane‐induced neuronal hyperactivity in the frontal cortex.

### 2.4 PERK suppression rescues cognitive impairment induced by sevoflurane anesthesia

**FIGURE 4:**
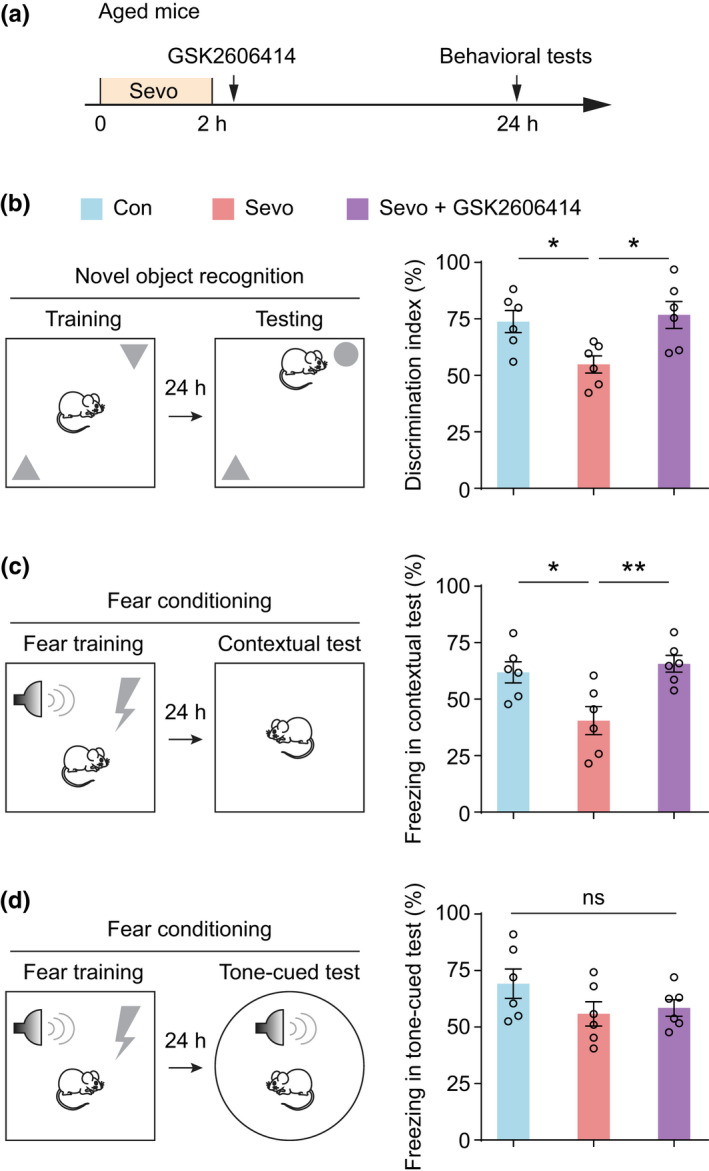
Inhibition of PERK ameliorates sevoflurane‐induced memory deficits in aged mice. (a) Experimental timeline for behavioral testing in aged mice after sevoflurane exposure. (b) Left, schematic of novel object recognition (NOR) training and testing. Right, discrimination ratio of aged mice with or without GSK2606414 treatment after sevoflurane anesthesia (*n* = 6 mice per group, *F* (2, 15) = 5.784, *p* = 0.0137). (c) Left, schematic of contextual fear conditioning and testing. Right, percentage of freezing duration in contextual fear test (*n* = 6 mice per group, *F* (2, 15) = 7.461, *p* = 0.0056). (d) Left, schematic of auditory‐cued fear training and testing. Right, percentage of freezing duration in tone‐cued fear test (*n* = 6 mice each group, *F* (2, 15) = 1.785, *p* = 0.2017). Throughout, each circle indicates the data from a single animal. Summary data are presented as mean ± *SEM*. ns, not significant; *p < 0.05, **p < 0.01 by one‐way ANOVA followed by Bonferroni's test

The frontal cortex plays important roles in higher cognitive functions such as learning and memory, planning and decision making. We next determined whether exposure of aged mice to sevoflurane impairs frontal dependent memory and whether inhibiting PERK may prevent such cognitive deficits. Specifically, aged WT mice received GSK2606414 following sevoflurane anesthesia and were subjected to a series of behavioral tests at 24 h (Figure 4a). In the novel object recognition (NOR) test, the preference to explore the novel object reflects the animal's recognition memory, which involves the frontal cortex (Kar & DiCarlo, 2021; Miller & Cohen, 2001). We separated the training and the test sessions by 24‐h intervals to examine the animals' long‐term memory. While the aged mice without sevoflurane anesthesia showed a preference toward the novel object during the NOR test, sevoflurane‐treated mice showed no such preference at 24 h after training (Figure 4b). This deficit in NOR memory was not observed in the group of aged mice treated with GSK2606414 during anesthesia recovery (Figure 4b).

In addition to the recognition memory, we also examined the animals' contextual and auditory‐cued fear memory after sevoflurane anesthesia. Compared with age‐matched controls, aged mice with sevoflurane anesthesia showed less freezing response in the contextual fear test (*p* = 0.0056) (Figure 4c). However, in the auditory‐cued fear test, we did not observe significant impairment after sevoflurane exposure (*p* = 0.2017) (Figure 4d). These results suggest that the brain regions involved in contextual fear learning, such as the hippocampus, may also be vulnerable to the disruption caused by sevoflurane anesthesia, consistent with previous reports (Le Freche et al., 2012). This deficit in fear memory was not observed in the group of mice that were administered GSK2606414 during anesthesia recovery (Figure 4c). Thus, we concluded that PERK suppression can rescue sevoflurane‐induced cognitive impairment in aged mice.

### 2.5 PERK suppression restores synaptic protein expression and Tau phosphorylation

**FIGURE 5:**
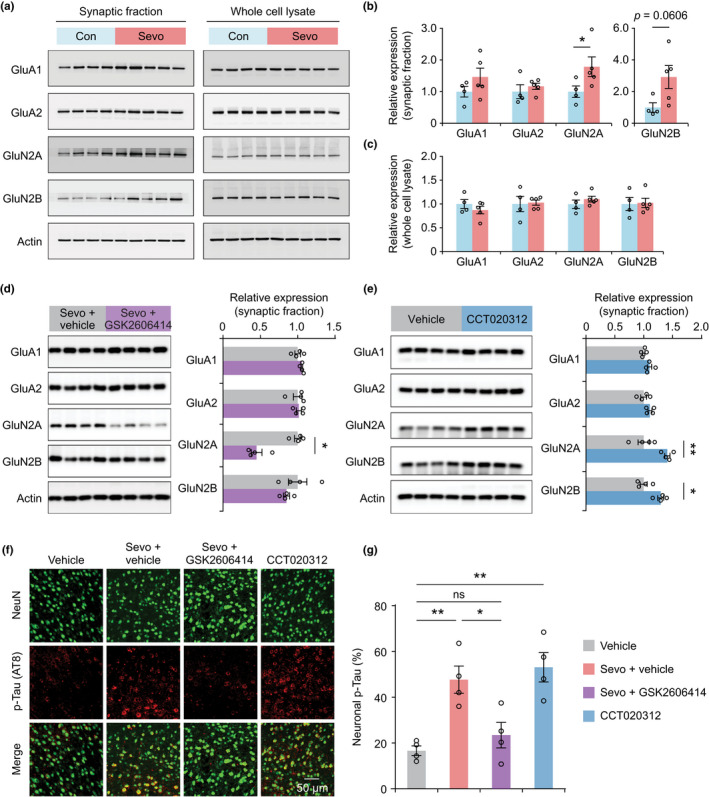
Inhibition of PERK restores synaptic GluN2A expression and Tau phosphorylation. (a) Synaptic fraction and whole‐cell lysates were generated from the frontal cortex of aged mice 4 h after sevoflurane anesthesia and probed with indicated antibodies by Western blotting. (b, c) Densitometric quantification of Western blots from the synaptic fraction (b) and whole‐cell lysates (c) (*n* = 4–5 mice per group). (d) Changes in synaptic protein expression in aged mice treated with GSK2606414 to inhibit PERK after sevoflurane anesthesia (*n* = 4 mice per group). (e) Changes of synaptic protein expression in non‐anesthetized control mice treated with CCT020312, a PERK activator (*n* = 4 mice per group). (f) Immunofluorescence staining of NeuN and phosphorylated tau (p‐Tau) (AT8) in the frontal cortex of aged mice 24 h after sevoflurane exposure. Scale bar, 50 µm. (g) Quantification of neuronal p‐Tau (*n* = 4 mice per group, *F* (3, 12) = 12.86, *p* = 0.0020). Throughout, each circle indicates the data from a single animal. Summary data are presented as mean ± *SEM*. ns, not significant; *p < 0.05, **p < 0.01 by unpaired, two‐tailed student's *t* test (b–e) or one‐way ANOVA followed by Bonferroni's test (g)

To investigate potential molecular mechanisms underlying sevoflurane‐induced frontal hyperexcitability, memory impairment and PERK suppression‐mediated protection in aged mice, we measured the amounts of various proteins in the cortex during the post‐anesthesia period, focusing on those involved in synaptic activity and function. Synaptosome and whole‐cell preparations were generated from the frontal cortex of aged mice at 4‐h post‐anesthesia, and protein concentrations were determined by Western blot analysis (Figure 5a). We found that postsynaptic glutamate NMDA receptor subunit GluN2A was significantly increased in synaptosomes from sevoflurane‐treated mice as compared to age‐matched controls (Figure 5a,b). The amount of GluN2B subunit also showed a trend of increase, although this trend did not reach statistical significance (*p* = 0.0606). There were no changes in glutamate AMPA receptor subunits (GluA1 and GluA2) 4 h after anesthesia. The amounts of GluN2A, GluN2B, GluA1, and GluA2 in the whole‐brain fraction remained unaltered (Figure 5a,c). These results indicate that sevoflurane anesthesia in aged mice causes increase in NMDA receptors at synapses, consistent with our finding of the increased NMDA receptor‐dependent Ca^2+^ activity in the frontal cortex (Figure 1f).

To investigate whether inhibiting PERK may restore normal synaptic protein expression in the frontal cortex, aged mice were administered GSK2606414 following sevoflurane anesthesia. At 4‐h post‐anesthesia, we found that mice treated with GSK2606414 showed a lower level of synaptic GluN2A expression in the fontal cortex as compared to age‐matched control mice treated with vehicle following sevoflurane exposure (Figure 5d), suggesting that inhibiting PERK with GSK2606414 prevents synaptic deficits during the post‐anesthesia period. In a separate experiment, we administered control mice CCT020312 (2 mg/kg, i.p.), a selective activator of PERK‐eIF2α signaling (Bruch et al., 2017) (Figure S3c,d). Consistent with the results above, activation of PERK with CCT020312 in aged mice without sevoflurane anesthesia increased the amount of GluN2A and GluN2B in synaptosome preparations (Figure 5e).

Previous studies have shown that sevoflurane anesthesia promotes tau protein phosphorylation in neonatal but not young adult mice (Lu et al., 2017; Tao et al., 2014; Yu et al., 2020), and the amounts of phospho‐Tau are inversely correlated with cognitive performance (Karikari et al., 2020). We examined the Tau protein phosphorylation in the frontal cortex of aged mice with or without sevoflurane exposure (Figure 5f,g). Four hours after the end of exposure, we observed a marked increase in phospho‐tau protein expression in frontal cortical neurons. Inhibiting PERK by GSK2606414 in post‐anesthesia mice significantly reduced the amount of phospho‐tau, whereas activating PERK with CCT020312 in non‐anesthetized mice resulted in an elevation of tau phosphorylation (Figure 5f,g). These results indicate that sevoflurane anesthesia promotes tau phosphorylation in aged brain and that PERK suppression reverses tau hyperphosphorylation induced by sevoflurane.

### 2.6 PERK suppression prevents sevoflurane‐induced synapse loss and cell death

**FIGURE 6:**
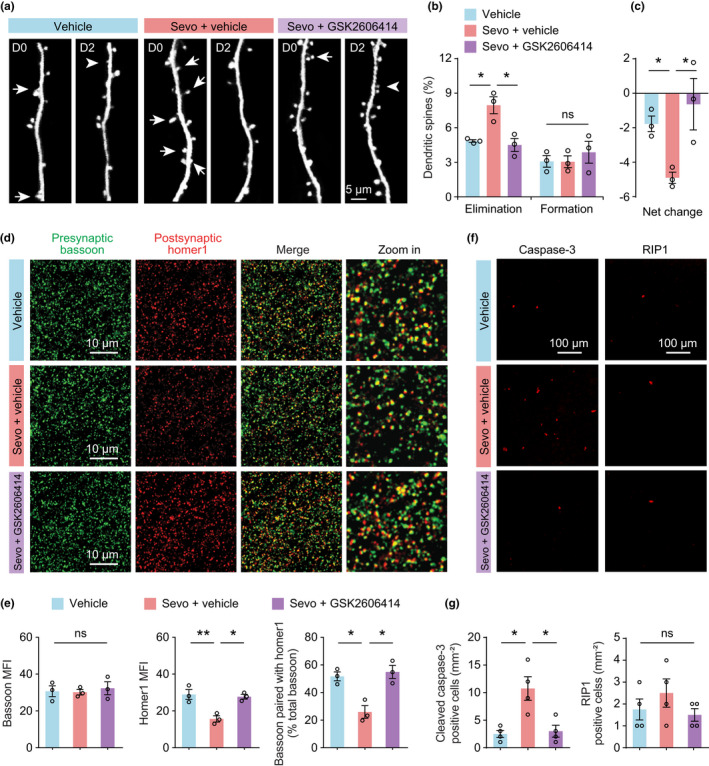
Inhibition of PERK rescues sevoflurane‐induced synapse loss and cell death in aged mice. (a) Representative images of transcranial two‐photon imaging of dendritic segments of L5 pyramidal neurons in the frontal cortex of aged mice. Arrows and arrowheads indicate individual spines that were eliminated or newly formed, respectively, over 2 days. (b) Spine formation and elimination over 2 days (*n* = 3 mice per group; elimination, *F* (2, 6) = 12.45, *p* = 0.0073; formation, *F* (2, 6) = 0.4612, *p* = 0.6511). (c) Net change in total spine number (*F* (2, 6) = 5.767, *p* = 0.0401). Scale bar, 5 µm. (d) Representative coronal sections of aged frontal cortex stained for the presynaptic marker bassoon and postsynaptic marker homer1 two days after sevoflurane exposure. Scale bar, 10 µm. (e) Quantification of images shown in d (*n* = 3 mice per group; bassoon, *F* (2, 6) = 0.1662, *p* = 0.8506; homer1, *F* (2, 6) = 13.92, *p* = 0.0056; bassoon paired with homer1, *F* (2, 6) = 14.18, *p* = 0.0053). MFI, mean fluorescence intensity. (f) Coronal sections of the aged frontal cortex stained for cell apoptosis marker cleaved caspase‐3 and cell necrosis marker RIP1 2 days after sevoflurane exposure. Scale bar, 100 µm. (g) Quantification of the number of cleaved caspase‐3 positive cells (*n* = 4 mice per group, *F* (2, 9) = 10.44, *p* = 0.0045) and RIP1 positive cells in the frontal cortex (*n* = 4 mice per group, *F* (2, 9) = 1.114, *p* = 0.369). Throughout, each circle indicates the data from a single animal. Summary data are presented as mean ± *SEM*. ns, not significant; *p < 0.05, **p < 0.01 by one‐way ANOVA followed by Bonferroni's test

The proper function of the frontal cortex is critically dependent on the integrity of synaptic connectivity in neuronal circuits. Previous studies have shown that general anesthesia can cause long‐lasting changes of synaptic plasticity in developing mice (Briner et al., 2010; Huang et al., 2016; Huang & Yang, 2015; Wenzel et al., 2021). Whether anesthesia alters synaptic plasticity in aged mice, however, remains unknown. Using transcranial two‐photon microscopy, we repeatedly imaged the apical dendrites of L5 pyramidal neurons in the frontal cortex of aged *Thy1*‐YFP‐H mice in vivo. Consistent with previous studies (Huang et al., 2020), we found that a small fraction of dendritic spines was formed and eliminated over 2 days in the aged brain (Figure 6a,b). In aged mice without sevoflurane anesthesia, 3.08 ± 0.12% of dendritic spines were formed and 4.85 ± 0.51% were eliminated over 2 days (524 spines, *n* = 3 mice). Exposure to sevoflurane significantly increased the rate of spine elimination (7.95 ± 0.74%, *p* < 0.05), but had no effect on the rate of spine formation (3.05 ± 0.51%, *p* = 0.46). As a result, an increased loss of total spine number was detected in the frontal cortex of aged mice within 2 days after sevoflurane anesthesia (Figure 6c). Notably, in aged mice administered GSK2606414 following sevoflurane anesthesia, the rate of dendritic spine elimination (4.51 ± 0.56%) was comparable to that of control mice without sevoflurane exposure. These findings indicate that inhibition of PERK activity prevents sevoflurane‐induced synapse loss in aged mice.

To further investigate the effects of PERK suppression on synapses, we examined the expression of the presynaptic scaffolding protein, bassoon, and the excitatory postsynaptic scaffolding protein, homer1, in the frontal cortex of aged mice 2 days after sevoflurane exposure (Figure 6d). Consistent with the loss of total spine number in the frontal cortex (Figure 6c), we found that the density of homer1 protein was significantly reduced after sevoflurane anesthesia (Figure 6d,e). There was no difference in the expression of presynaptic protein bassoon between mice with and without sevoflurane anesthesia. Administration of GSK2606414 after sevoflurane anesthesia restored the expression of homer1 in the frontal cortex of aged mice. These results support the finding that PERK inhibition prevents sevoflurane‐induced synapse loss in aged mice.

It is well acknowledged that prolonged exposure to general anesthesia can increase cell apoptosis (Dong et al., 2009; Jevtovic‐Todorovic et al., 2003; Xie et al., 2006; Zhang et al., 2010). The rescue of synapse loss by PERK suppression suggests that anesthesia‐induced cell loss may also be rescued. To test this, we examined cell death in the fontal cortex of aged mice 2 days after sevoflurane exposure. Cell apoptosis and necrosis were assessed by the immunostaining of cleaved caspase‐3 and RIP1, respectively (Figure 6f). We found that exposure of aged mice to sevoflurane for 2 h resulted in a significant increase in cell apoptosis (Figure 6f,g), particularly for neurons (Figure S4), but had no effect on necrosis (Figure 6f,g), consistent with the notion that the long‐lasting activation of PERK‐eIF2α signaling promotes the transcription and translation of genes related to cell apoptosis (Hetz & Saxena, 2017). Administration of GSK2606414 to inhibit PERK in aged mice prevented sevoflurane‐induced cell apoptosis (Figure 6f,g).

## 3 DISCUSSION

Cognitive impairment is an important domain of delirium syndrome, which often occurs within days after anesthesia and surgery in elderly patients. The objective of the present study was to investigate the potential mechanisms by which sevoflurane induces cognitive impairment in aged mice. We showed that sevoflurane anesthesia caused an increase in neuronal activity in the frontal cortex of aged mice, which lasted for at least 24 h after the end of exposure. In parallel, frontal cortical neurons exhibited increased phosphorylation of PERK and eIF2α, the markers of UPR activation. Genetic deletion or pharmacological inhibition of PERK prevented anesthesia‐induced frontal neuronal hyperactivity. Moreover, inhibiting PERK in aged mice prevented sevoflurane‐induced cognitive impairment and synapse loss. Together, our findings highlight UPR suppression as a potential strategy to protect the aged brain from the deleterious effects caused by anesthesia.

Mounting evidence suggests that general anesthesia may have undesired effects on brain function, and the elderly are particularly susceptible to post‐anesthesia delirium and associated cognitive decline (Lipowski, 1989; Sprung et al., 2017). The incidence of postoperative delirium is at least twice higher in individuals older than 65 years compared with younger adults (Alalawi & Yasmeen, 2018). Similar age‐dependent changes in behavior have also been observed in animals subjected to anesthesia and surgery (Kilicaslan et al., 2013; Liufu et al., 2020; Wiklund et al., 2009). Aging causes a number of changes in the brain that may contribute to its susceptibility to the stresses of anesthesia and surgery. It has been shown that age‐associated neuroinflammation (Luo et al., 2019) and neurodegeneration (Xu et al., 2019) become worse after anesthesia and surgery. In the older brain, aging cells have decreased levels of certain ER proteins, including protein chaperones that are important for ensuring proper protein folding and degradation of misfolded proteins (Paz Gavilan et al., 2006), suggesting that the older brain is more vulnerable to anesthesia‐induced ER stress. Indeed, we observed a prolonged activation of UPR in aged but not young mice after sevoflurane anesthesia, which may contribute to abnormal increases in tau phosphorylation, neuronal hyperactivity, and cognitive impairment. Supporting this hypothesis, inhibition of PERK, a key component of UPR, prevented neurological sequelae induced by sevoflurane.

General anesthetics are powerful modulators of neuronal activity (Rudolph & Antkowiak, 2004; Yamakura et al., 2001). Previous studies have shown that cortical activity is largely suppressed during the course of anesthesia and recovers afterward (Huang et al., 2016; Zhou et al., 2021). Consistent with these studies, our data showed that exposure of both young and aged mice to 3% sevoflurane caused a substantial reduction in somatic Ca^2+^ transients in the mouse frontal cortex. After the end of exposure, the level of pyramidal neuronal activity in young adult mice was quickly increased and restored to the pre‐anesthesia baseline. The level of neuronal activity in aged mice, however, was overtly increased, exceeding the baseline for at least 24 h. As neuronal activity is important for learning and memory, this dysregulation of Ca^2+^ activity in the frontal cortex may contribute to post‐anesthesia cognitive impairment. Interestingly, similar to aged animals, neonatal rodents exposed to sevoflurane anesthesia also exhibited an increase in ER stress‐related proteins and neuronal hyperactivity (Shen et al., 2018; Xie et al., 2020; Yang et al., 2020).

Increasing evidence suggests that neuronal hyperactivity is an early functional hallmark of neurodegeneration, particularly in ADRD. Using functional magnetic resonance imaging, neuronal network hyperactivity has been detected in human patients with mild cognitive impairment (Dickerson et al., 2005), as well as in presymptomatic individuals carrying the genetic mutations associated with AD (Quiroz et al., 2010). In transgenic AD animals, it was reported that a subset of cortical neurons near amyloid plaques exhibited an increase in the frequency of spontaneous Ca^2+^ transients (Busche et al., 2008). Similar increases in neuronal activity were detected in the mouse cortex and hippocampus before the formation of amyloid plaques (Bai et al., 2017; Busche et al., 2012). In AD, excessive neuronal activity has been associated with reduced neural plasticity, synaptic deficits, and failure to encode new memory. For instance, a recent in vivo imaging study in 3‐month‐old AD mice showed that dendritic spines in cortical pyramidal neurons displayed a decrease in activity and size after abnormal long‐duration dendritic calcium transients (Bai et al., 2017). Similarly, we found that in aged mice with sevoflurane anesthesia, the abnormal increase in Ca^2+^ transients in cortical pyramidal neurons was followed by an increase in dendritic spine elimination. These findings suggest that delirium may have neuropathogenic features in common with AD. Indeed, postoperative delirium has been shown to accelerate cognitive decline in AD patients (Fong et al., 2009).

A potential cause of neuronal hyperactivity in post‐anesthesia mice may be enhanced synaptic NMDA receptor function. Our examination of synaptic proteins revealed an upregulation of synaptic GluN2A subunits in the aged frontal cortex 4 h after sevoflurane exposure, consistent with the finding of increased NMDA receptor‐dependent Ca^2+^ spikes in pyramidal neurons during anesthesia recovery. Supporting the role of GluN2A in neuronal hyperactivity, restoring synaptic NMDA receptor expression by inhibiting PERK reversed neuronal hyperactivity induced by sevoflurane. GluN2A and GluN2B are the most abundant NMDA receptor subunits expressed in the mammalian brain (Paoletti et al., 2013). GluN2A‐containing NMDA receptors are highly expressed in the adult hippocampus and neocortex and enriched in postsynaptic density. It was previously shown that exposure of neonatal rats to sevoflurane increases the expression of GluN1 and GluN2B in the frontal cortex (Zhang et al., 2016). Administration of memantine, a NMDA receptor antagonist, rescued learning and memory dysfunction induced by developmental sevoflurane exposure (Wang et al., 2016). Future studies are needed to examine whether drug treatment that lowers NMDA receptor activity after sevoflurane anesthesia might improve cognitive function in aged animals.

Our study highlights the role of PERK‐eIF2α signaling in mediating anesthesia‐induced neuropathology in aged mice. PERK‐dependent phosphorylation of eIF2α constitutes one of the three branches of UPR, a signaling pathway that is activated by ER stress and protects cells by restoring normal proteostasis (Hetz & Saxena, 2017). It is normally believed that short‐term activation of UPR is protective in maintaining neuronal functioning, whereas long‐term activation can result in synaptic deficits and cell death (Freeman & Mallucci, 2016; Kim et al., 2006). Indeed, increased phosphorylation of PERK and eIF2α has been reported in studies of neurodegenerative and neuropsychiatric diseases (Hetz & Saxena, 2017; Hughes & Mallucci, 2019). For instance, neonatal rats repeatedly exposed to sevoflurane displayed an upregulation of hippocampal PERK‐eIF2α signaling and performance impairment in the Morris water maze, a task that involves hippocampal function (Shen et al., 2018). Inhibition of PERK phosphorylation in neonatal mice attenuated sevoflurane‐induced neuronal apoptosis and BACE‐1 expression (Liu et al., 2017). In young adult mice, we found that a single exposure to sevoflurane anesthesia had no apparent effects on PERK phosphorylation in the frontal cortex. However, exposure of aged mice to sevoflurane caused a protracted activation of PERK‐eIF2α signaling, and PERK suppression protected aged mice from anesthesia‐induced abnormal GluN2A upregulation, neuronal hyperactivity, synapse loss, and memory impairment.

In summary, we showed that the anesthetic sevoflurane caused neuronal hyperactivity and synapse loss in the frontal cortex of aged mice, which was mediated by the activation of neuronal PERK‐eIF2α signaling. Suppression of PERK activity in post‐anesthesia mice restored the expression of synaptic NMDA receptors and prevented sevoflurane‐induced neuronal hyperactivity, synapse loss and memory impairment. PERK suppression may serve as a potential therapeutic strategy to prevent perioperative neurocognitive disorder in the elderly.

## 4 EXPERIMENTAL PROCEDURES

### 4.1 Animals

Transgenic *Thy1*.*2*‐GCaMP6slow (line 1) mice were used for Ca^2+^ imaging experiments (Cichon et al., 2020). *Perk* ^loxP^ (Stock No: 023066) mice were purchased from the Jackson Laboratory and used for the selective knockout of PERK. *Thy1*‐YFP‐H mice (Stock No: 003782) were used for dendritic spine imaging experiments. Young adult (4 months) and aged (>18 months) mice were group‐housed in the animal facility at Columbia University Medical Center and randomly assigned to different treatment groups. Both male and female mice were used. All animal procedures were approved by Institutional Animal Care and Use Committee (IACUC) at Columbia University (Protocol: AABN7553) and carried out in accordance with the National Institutes of Health (NIH) guidelines for animal care and use. The manuscript was written according to ARRIVE.

### 4.2 Sevoflurane anesthesia

Experimental mice were exposed to sevoflurane anesthesia for 2 h. During this procedure, mice were placed in a closed acrylic chamber, receiving 3% sevoflurane in 30% oxygen. During anesthesia, a heating pad was used to maintain the animal's body temperature at approximately 37°C. Control mice received no treatment but were placed in the same acrylic chamber.

### 4.3 Drug administration

MK801 (M107, Sigma‐Aldrich) was dissolved in artificial cerebrospinal fluid (ACSF) to the final concentration (100 µM) (Chen et al., 2019). In head‐fixed animals, MK801 (0.2 µl per animal) was locally injected into layer 5 of the mouse frontal cortex using a glass microelectrode. Because small molecules diffuse rapidly in the cortex, we estimated that the drug concentration was reduced ~10 times in the imaged cortical region, such that the final effective concentration would be ~10 μM. GSK2606414 (516535, Millipore Sigma) was dissolved in distilled water containing 0.5% hydroxypropylmethyl cellulose and 0.1% Tween‐80 at pH 4.0. For PERK inhibition, GSK2606414 (50 mg/kg) was administered to animals via oral gavage. CCT020312 (324879, Millipore Sigma) was dissolved in DMSO as stock solutions and diluted in 0.9% saline. To activate PERK, CCT020312 (2 mg/kg) was delivered by intraperitoneal injection.

### 4.4 Two photon Ca^2+^ imaging and data analysis

In vivo Ca^2+^ imaging was performed in head‐restrained mice before, during, and after sevoflurane anesthesia. Surgical preparation for imaging awake, head‐restrained mice has been described in detail in previous publications (Yang et al., 2013). In brief, mice were anesthetized with an intraperitoneal injection of 100 mg/kg ketamine and 15 mg/kg xylazine. After a midline skin incision, a head holder consisting of two metal bars was attached to the animal's skull using glue and dental cement. After the cement was completely dry, a cranial window was created over the frontal association cortex (2.8 mm anterior of bregma and 1.0 mm lateral from the midline) as previously described (Cichon et al., 2017). The completed cranial window was covered with silicone elastomer. Mice were given at least 24 h to recover from the surgery‐related anesthesia. Later, mice with the head holder were habituated to the mounting device a few times (10 min each) to minimize potential stress related to head restraining and imaging.

To image Ca^2+^ activity in the cortex of awake mice, the head holder was screwed to a metal plate. The silicon elastomer was removed, and the cranial window was cleaned with ACSF. The head‐fixed animal together with the metal plate was then placed on the stage of microscope. In vivo Ca^2+^ imaging was performed using a Scientifica two‐photon system equipped with a Ti:Sapphire laser (Vision S, Coherent). The average laser power on the sample was ~20 to 30 mW. Images were collected using a 25× water‐immersion objective (N.A. 1.05) at a frame rate of 1 Hz and a size of 512 × 512 pixels. Image acquisition was performed using ScanImage software (Vidrio Technologies).

Images were analyzed post hoc using the NIH ImageJ software as well as toolboxes and custom code in MATLAB as previously described (Adler et al., 2019; Zhou et al., 2021). Δ*F*/*F*_0_ was calculated as (*F*–*F*_0_)/*F*_0_, where *F*_0_ is the baseline fluorescence signal averaged over a 6‐s period corresponding to the lowest fluorescence signal during the recording period.

### 4.5 Viral injection

To conditionally knockout PERK in frontal cortical neurons, recombinant adeno‐associated virus (AAV) encoding Cre under the hSyn promoter (AAV9‐hSyn‐Cre; Addgene, 105553) was bilaterally injected into the frontal cortex of PERK^fl/fl^ mice and PERK^+/+^ mice (control). For Ca^2+^ imaging of pyramidal neurons in the frontal cortex of PERK^fl/fl^ and PERK^+/+^ mice, GCaMP6s was virally expressed under the CaMKII promoter (AAV9‐CaMKII‐GCaMP6s; Addgene, 107790). For each vector, 0.2 μl of AAV (>1 × 10^13^ GC/ml titer) were diluted 5× in ACSF and slowly injected (Picospritzer III; 12 p.s.i., 5 ms, 0.5 Hz) over 10 min into the frontal cortex using a glass microelectrode around the coordinates of AP + 2.80 mm, ML 1.0 mm, DV 0.5 mm. During injection, a heating pad was used to maintain the animals’ body temperature at ~37°C. After injection, the scalp was sutured, and mice were returned to their home cages. Mice infected with AAV were prepared for head‐fixation and imaging 3–4 weeks following AAV microinjection.

### 4.6 Novel object recognition

All behavioral experiments were performed blind to treatment groups. Mice were tested for novel object recognition (NOR) in an acrylic chamber (25 × 25 × 25 cm dimensions). Behavior was videotaped, and images were analyzed with ANY‐maze behavior tracking software (Stoelting). Mice were first habituated to the environment for 10 min with no objects in the chamber. During NOR training, mice were placed in the chamber for 10 min with two identical objects placed in the opposite corners of the chamber. After training, mice were returned to their home cages. Twenty‐four hours later, mice were returned to the chamber for 10 min where one familiar object was replaced by a novel object that was different in both shape and texture. Measures of explorative interaction were taken as the amount of time that the animal spent with its head and nose oriented toward and within 2 cm of the object. Other types of interactions such as the animal accidentally touching, sitting, or standing on the object were not considered exploratory activity. Discrimination index was calculated as the time spent interacting with the novel object divided by the total time spent exploring both objects.

### 4.7 Fear conditioning

Fear conditioning was conducted in a fear conditioning chamber (Maze Engineers). The ANY‐maze software (Stoelting) was used to control the delivery of tones and foot shocks. Mice were placed in the cleaned chamber for 2 min before they were presented with an auditory cue (400 Hz, 80 dB) for 30 s. A mild foot shock (0.5 mA) was delivered during the last 2 s of the tone presentation and co‐terminated with the tone. A total of 3 trials were repeated with a 15‐s intertrial interval. After fear conditioning, mice were returned to their home cages. The next day, contextual fear memory was tested by returning mice to the same chamber for 5 min without applying the shock. Fear memory was calculated as the percent of time that mice spent on freezing during the total time in chamber. Auditory‐cued fear memory was tested by placing mice to a different chamber and presenting the auditory cue for 1 min. Fear memory was measured by the percent of time that the animals spent on freezing during the tone presentation.

### 4.8 Immunohistochemistry

Mice were deeply anesthetized and perfused with 4% paraformaldehyde. Brain samples were dehydrated with 30% sucrose overnight and sectioned into 80‐μm coronal slices using a Leica VT1000 S vibratome. The brain slices were rinsed twice in phosphate‐buffered saline (PBS), blocked with goat serum at room temperature for 1 h, and then incubated at 4°C for 48 h with the primary antibodies: rabbit anti‐phospho‐eIF2a (CST, 3398S, 1:200), rabbit anti‐PERK (CST, 5683S, 1:200), mouse anti‐phospho‐Tau AT8 (Invitrogen, MN1020B, 1:400), rabbit anti‐NeuN (Abcam, ab177487, 1:500), mouse anti‐NeuN (Abcam, ab104224, 1:500), rabbit anti‐homer1 (Invitrogen, PA521487, 1:200), mouse anti‐bassoon (Invitrogen, MA120689, 1:200), rabbit anti‐cleaved caspase‐3 (CST, 9661S, 1:300), and rabbit anti‐RIP1 (CST, 3493S, 1:100). Brain slices were washed twice in PBS and incubated for 1 h with the secondary antibody: anti‐rabbit‐Alexa Fluor 594 or anti‐mouse‐Alexa Fluor 488. After washes, sections were mounted with DAPI solution. Images were captured with a confocal microscope (Nikon, Japan) and analyzed using ImageJ software.

### 4.9 Western blotting

Mice were euthanized and frontal cortex lysates were extracted using the cocktail containing protease and phosphatase inhibitor (Thermo Scientific, 78442). Lysates were stored at −80°C when not used immediately. Synaptosome was isolated using the Syn‐PER™ synaptic protein extraction reagent (Thermo Scientific, 87793). Protein quantification was performed using Pierce BCA protein assay kit (Thermo Scientific, 23227). Lysates containing 10 μg protein were separated using SDS‐polyacrylamide gel electrophoresis and transferred to a polyvinylidene difluoride membrane. The membranes were washed twice in PBS with Tween 20 (PBST), blocked with 5% bovine serum albumin, and then incubated at 4°C overnight with primary antibodies: rabbit anti‐GluA1 (CST, 13185S, 1:1000), rabbit anti‐GluA2 (CST, 13607S, 1:1000), rabbit anti‐GluN2A (CST, 4205S, 1:1000), rabbit anti‐GluN2B (CST, 14544S, 1:1000), rabbit anti‐PERK (CST, 5683S, 1:1000), rabbit anti‐phospho‐PERK (CST, 3179S, 1:1000), rabbit anti‐eIF2α (CST, 5324S, 1:1000), rabbit anti‐phospho‐eIF2α (CST, 3398S, 1:1000), mouse anti‐actin (CST, 3700S, 1:1000), or rabbit anti‐tubulin (CST, 5568S, 1:1000). The membranes were washed in PBST and incubated for 2 h with secondary antibodies: HRP‐linked anti‐rabbit IgG (CST, 7074S, 1:1000) or anti‐mouse IgG (CST, 7076S, 1:1000). Protein bands were visualized using an imaging system (Image Quant LAS‐4000). Protein expression differences were measured using Image Quant software.

### 4.10 In vivo imaging of dendritic spines and data analysis

The surgical procedure for transcranial two‐photon imaging of dendritic spines has been described previously (Yang et al., 2010). In brief, the animal was anesthetized, and a custom‐made head plate was glued to the skull with the central opening over the frontal association cortex. A thinned‐skull window (200 µm in diameter, 20 µm in thickness) was created by using a dental drill and a microsurgical blade. The anesthetized mice were then placed on the stage of a Scientifica two‐photon microscope with the laser tuned to the optimal excitation wavelength for YFP (920 nm). Using a 25× water‐immersion objective (1.05 N.A.), five to eight stacks of image planes within a depth of 100 µm from the pial surface were collected at a digital zoom of 1 to 4×. After image acquisition, the head plate was gently detached from the skull, and the scalp was sutured with 6–0 thread. The animals were returned to their home cages until the next view.

Data analysis was performed using NIH ImageJ software. The same dendritic segments were identified from three‐dimensional image stacks taken at both time points. The number and location of dendritic protrusions were identified in each view. Filopodia were identified as long, thin structures without enlarged heads, and the rest of the protrusions were classified as spines. Spines were considered the same between two views based on their spatial relationship to adjacent spines and other landmarks. Spines in the second view were considered different if they were more than 0.7 µm away from their expected positions based on the first view. The formation and elimination rates of spines were measured as the number of spines formed or eliminated divided by the number of spines existing in the first view.

### 4.11 Statistics

All data were presented as mean ± *SEM*. Prism software (GraphPad 7.05) was used to conduct the statistical analysis. Tests for differences between two groups were performed using two‐tailed Student's *t* test. Multiple‐group comparison was performed using one‐way ANOVA followed by Bonferroni's post hoc tests. Significant levels were set at *p* < 0.05.

## CONFLICT OF INTEREST

The authors declare that they have no competing interests. Dr. Zhongcong Xie provided consulting service to Baxter, Shanghai 9th and 10th hospitals.

## AUTHOR CONTRIBUTIONS

K.C., Q.H., Z.X., and G.Y. designed research studies. K.C. performed in vivo imaging and behavior experiments. Q.H. and R.G. performed biochemical experiments. K.C., Q.H., R.G., Z.X., and G.Y. analyzed the data. K.C., Q.H., R.G., J.S., Z.X., and G.Y. wrote and revised the manuscript.

## Supporting information

Figures S1–S4

## Data Availability

The data that support the findings of this study are available from the corresponding author upon reasonable request.
