# Supplementary material for: Inhibition of unfolded protein response prevents post‐anesthesia neuronal hyperactivity and synapse loss in aged mice
Source: Aging Cell. 2022 Mar 17;21(4):e13592. doi: 10.1111/acel.13592 (PMC9009124; doi:10.1111/acel.13592)
Supplement: Supplementary file 1 — Figures S1–S4 [file ACEL-21-e13592-s001.docx]

**Supporting Information**

**Inhibition of unfolded protein response prevents post-anesthesia neuronal hyperactivity and synapse loss in aged mice**

**Chen et al.**


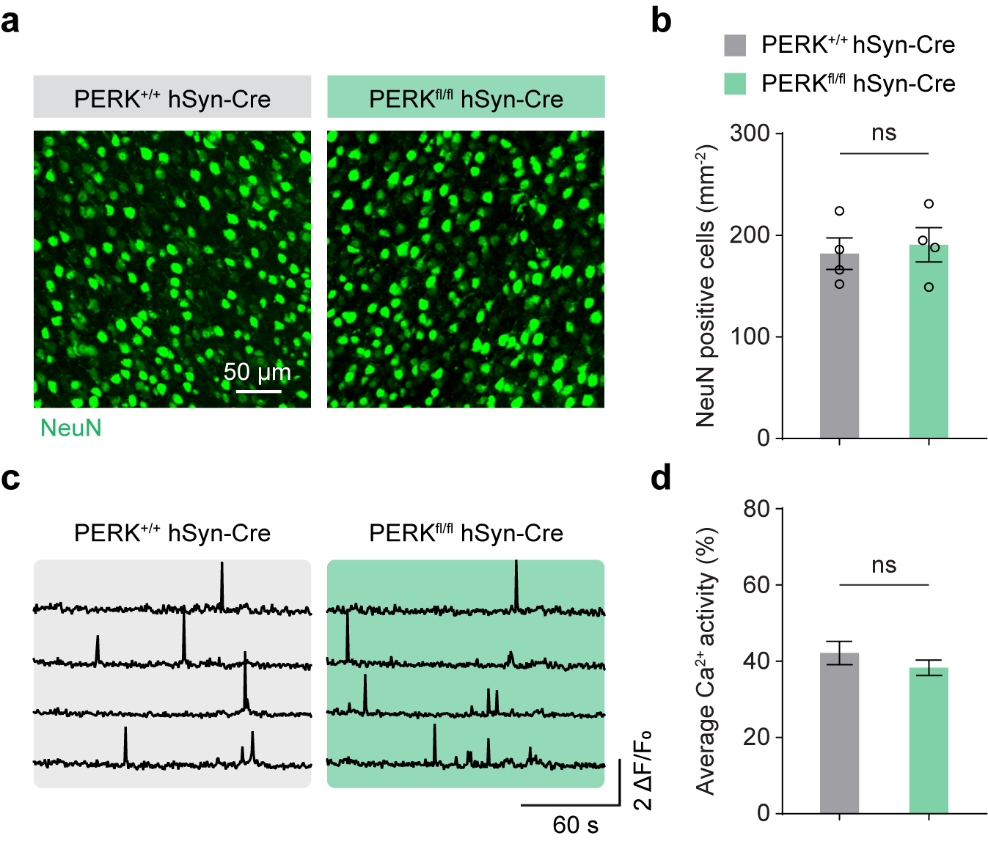


**Figure S1 Genetic deletion of PERK in frontal cortical neurons has no effect on neuronal number and baseline Ca^2+^ activity**.

(**a**) NeuN staining in the frontal cortex of aged mice with (PERK^fl/fl^ hSyn-Cre AAV) and without (PERK^+/+^ hSyn-Cre AAV) neuronal PERK deletion. Scale bar, 50 µm. (**b**) Quantification of NeuN^+^ cell number in the frontal cortex (*n* = 4 mice per group; *t*^­^_6_ = 0.381, *p* = 0.716). (**c**) Representative Ca^2+^ traces in the frontal cortex of aged mice with and without PERK deletion. (**d**) Average integrated Ca^2+^ activity of pyramidal neurons in aged mice with and without PERK deletion (*n* = 4 mice per group; *t*^­^_497_ = 1.057, *p* = 0.291). Summary data are presented as mean ± s.e.m. ns, not significant by unpaired, two-tailed student’s *t* test.


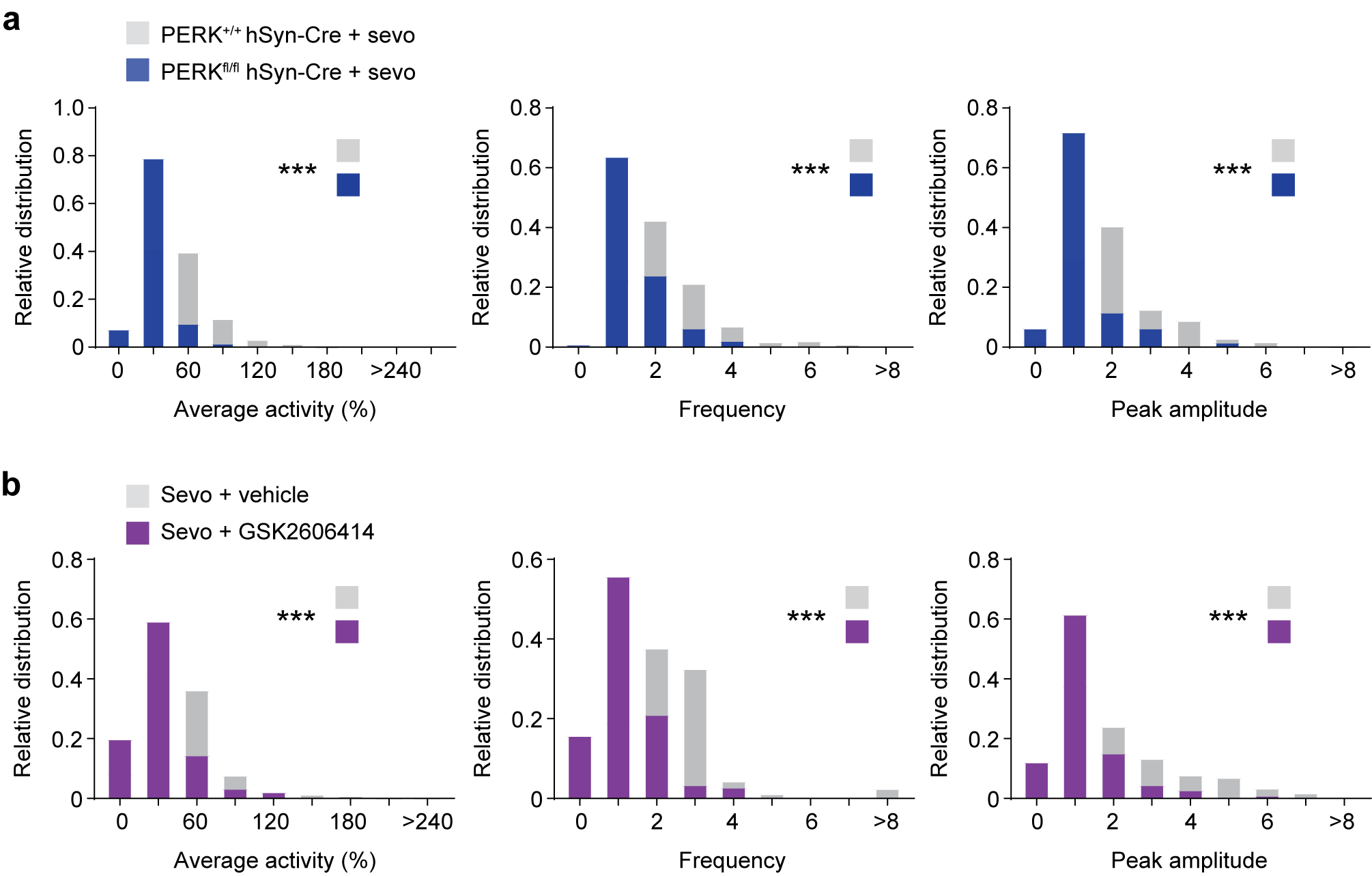


**Figure S2 Inhibition of PERK attenuates sevoflurane-induced neuronal hyperactivity in aged mice**.

(**a**) Distribution of average integrated activity, frequency, and peak amplitude of Ca^2+^ transients in aged PERK^+/+^ hSyn-Cre and PERK^fl/fl^ hSyn-Cre mice 4 h post-anesthesia (*n* = 4 mice per group; average activity, *t*^­^_433_ = 9.184, *p* < 0.0001; frequency, *t^­^*_433_ = 6.489, *p* < 0.0001; amplitude, *t*^­^_433_ = 8.538, *p* < 0.0001) (**b**) Distribution of average integrated activity, frequency, and peak amplitude of Ca^2+^ transients in aged mice treated with vehicle or GSK2606414 after sevoflurane anesthesia (*n* = 4 mice each group; average activity, *t*_434_ = 5.546, *p* < 0.0001; frequency, *t^­^*_432_ = 8.602, *p* < 0.0001; amplitude, *t*_420_ = 6.645, *p* < 0.0001). ****p* < 0.001 by unpaired, two-tailed student’s *t* test.


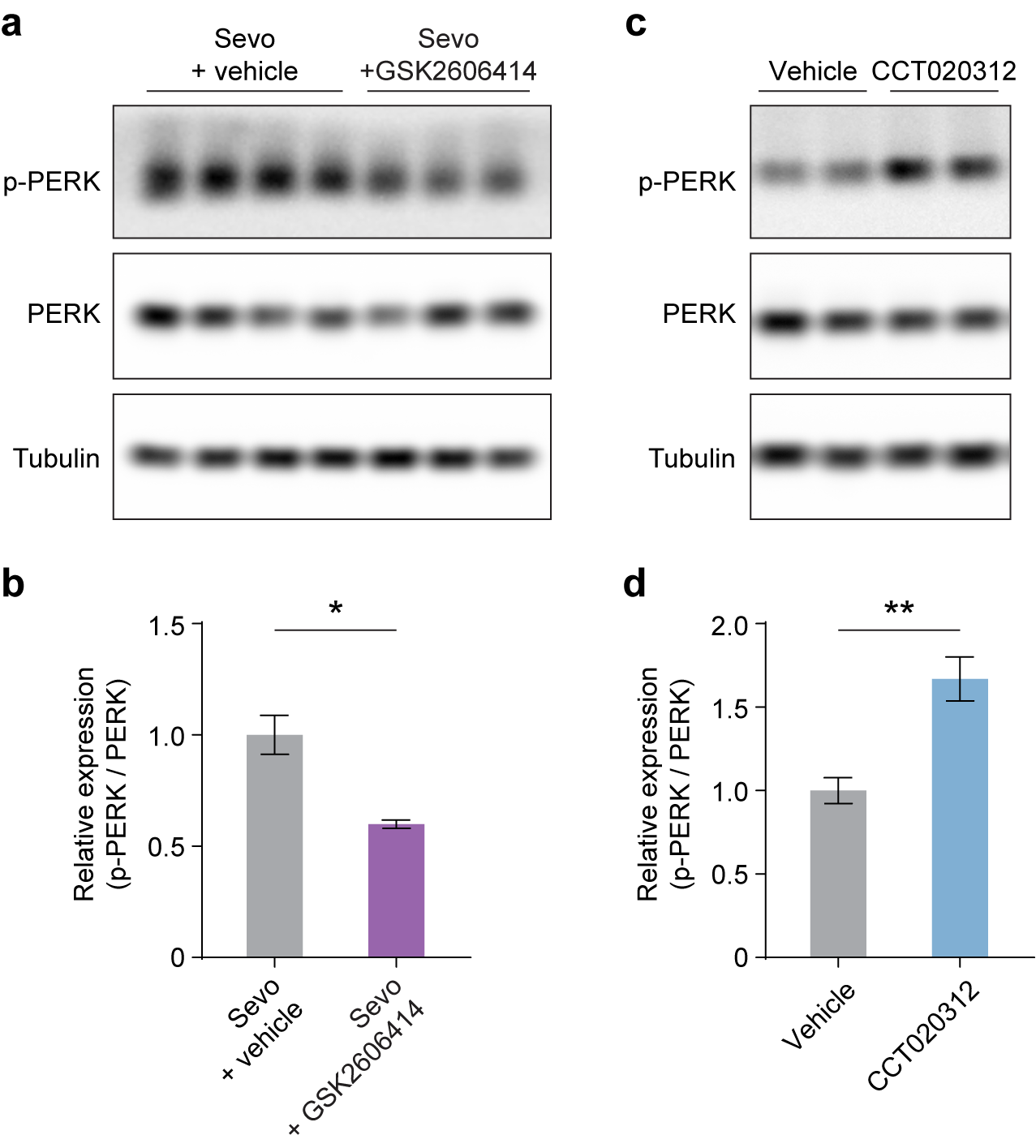


**Figure S3 Verification of PERK inhibition or activation by pharmacological treatment**.

(**a**) Western blot analysis of PERK expression in the frontal cortex of aged mice 4 h after sevoflurane exposure. (**b**) Densitometric quantification of the ratio of p-PERK to PERK in the frontal cortex of aged mice administered vehicle or GSK2606414, a highly selective PERK inhibitor, following sevoflurane exposure (Sevo + vehicle, *n* = 4 mice; Sevo + GSK2606414, *n* = 3 mice; *t*^­^_5_ = 3.827, *p* = 0.012). (**c**) Western blot showing PERK expression in the frontal cortex of aged mice without sevoflurane exposure. (**d**) Densitometric quantification of the ratio of p-PERK to PERK in the frontal cortex of aged mice administered vehicle or CCT020312, a selective PERK activator (vehicle, *n* = 4 mice; CCT020312, *n* = 3 mice; *t*^­^_5_ = 3.827, *p* = 0.005). Summary data are presented as mean ± s.e.m. **p* < 0.05, ***p* < 0.01 by student’s *t* test.


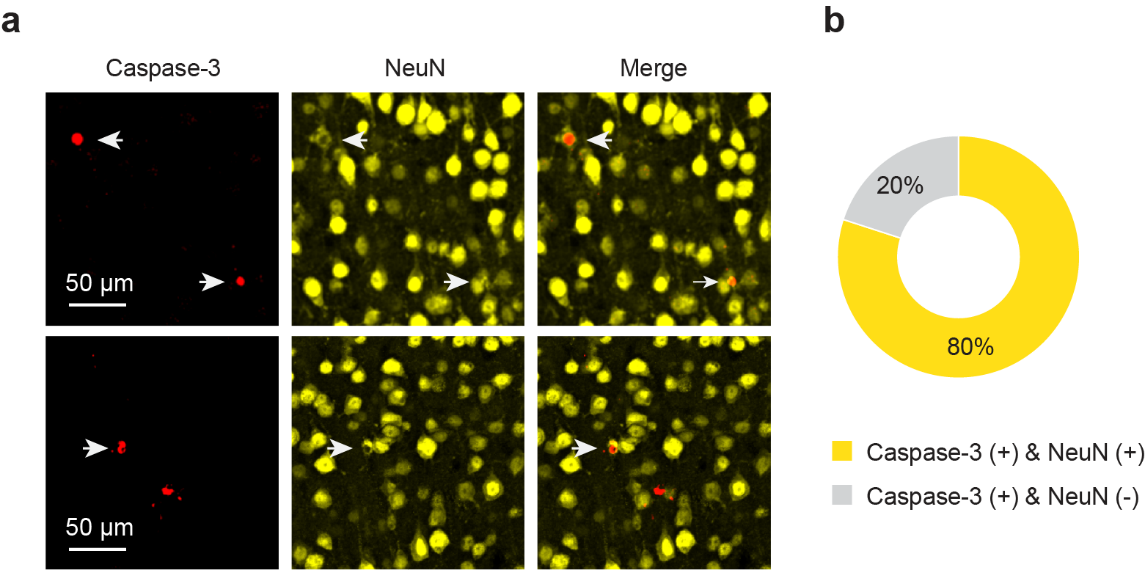


**Figure S4 Sevoflurane induces neuronal apoptosis in the frontal cortex of aged mice**.

(**a**) Representative coronal sections of the aged frontal cortex stained for a cell apoptosis marker cleaved caspase-3 (red) and a neuronal marker NeuN (yellow) two days after sevoflurane exposure. Arrowhead indicates the colocalization of capases-3 and NeuN. Scale bar, 50 µm. (**b**) Quantification of data shown in **a**. The majority of cleaved caspase-3 positive cells are colabeled with NeuN.
